# Environmental impact of dietary patterns in 10 European countries; a cross-sectional analysis of nationally representative dietary surveys

**DOI:** 10.1093/eurpub/ckae088

**Published:** 2024-05-22

**Authors:** Ricardo Alves, Julian Perelman, Kiara Chang, Christopher Millett

**Affiliations:** NOVA National School of Public Health, Public Health Research Centre, Comprehensive Health Research Center, CHRC, NOVA University Lisbon, Lisbon, Portugal; NOVA National School of Public Health, Public Health Research Centre, Comprehensive Health Research Center, CHRC, NOVA University Lisbon, Lisbon, Portugal; School of Public Health, Imperial College London, Public Health Policy Evaluation Unit, London, UK; NOVA National School of Public Health, Public Health Research Centre, Comprehensive Health Research Center, CHRC, NOVA University Lisbon, Lisbon, Portugal; School of Public Health, Imperial College London, Public Health Policy Evaluation Unit, London, UK

## Abstract

**Background:**

Changing dietary patterns is essential to reducing the substantial environment impact of agriculture and food production systems. We performed a cross-country comparison of dietary patterns and their associated environmental impact in Europe, including by sociodemographic factors.

**Methods:**

We analyzed pooled cross-sectional dietary records collected during 2010–18 from 10 European countries using the European Food Safety Authority (EFSA) Comprehensive European Food Database (16 508 adults; aged 18–79 years). Each food consumed was mapped to the corresponding environmental impact data using the SHARP Indicators Database, which provides greenhouse gas emission (GHGE) and land use (LU) values of approximately 900 foods. Total diet-associated environmental impact was calculated for each person and averaged across multiple days. Multivariable linear regression models were used to compare diet-associated GHGE and LU between population subgroups (gender, age, education and diet type) with country-level fixed effects.

**Results:**

The mean dietary GHGE and LU per capita ranged from 4.0 kgCO_2_/day and 5.0 m^2^*year/day in Spain to 6.5 kgCO_2_eq/day and 8.2 m^2^*year/day in France. Diet-related GHGE and LU (per kg/food) were lower among females (2.6 kgCO_2_eq/day, *B* = −0.08, *P* < 0.01; 3.2 m^2^*year/day, *B* = −0.11, *P* < 0.01), older population aged 66–79 (2.6 kgCO_2_eq/day, *B* = −0.03, *P* < 0.01; 3.4 m^2^*year/day, *B* = −0.4, *P* < 0.01), people following vegetarian diets (1.7 kgCO_2_eq/day, *B* = −0.07, *P* < 0.01; 2.0 m^2^*year/day, *B* = −0.07, *P* < 0.01), and higher among individuals with secondary education (2.7 kgCO_2_eq/day, *B* = 0.05, *P* < 0.01; 3.6 m^2^*year/day, *B* = −0.05, *P* < 0.01).

**Conclusions:**

Environmental footprints vary substantially across countries, dietary patterns and between different sociodemographic groups in Europe. These findings are crucial for the development of country-specific food policies aimed at promoting environmentally sustainable diets.

## Introduction

Achieving more sustainable and healthy diets is a common intergovernmental goal to address the rapidly growing impact of climate change. In recent years, there has been a growing consensus among scientists and policymakers regarding the potential of dietary changes to mitigate the environmental pressure associated with the food and agriculture system.^1–4^ Food production, industrial food processing, storage and retailing significantly contributes to greenhouse gas emissions (GHGE), water scarcity, land degradation and biodiversity loss.^4^^,^^5^ Greenhouse gas emissions are a notable consequence, with the food sector alone responsible for 26% of these emissions.^6^ Agriculture, in particular, accounts for approximately 70% of global freshwater withdrawals,^7^ while nearly half of all habitable land is utilized for agricultural purposes.^8^ The rearing of livestock for meat and dairy production involves extensive land use (LU), which can contribute to deforestation and habitat destruction. Moreover, the methane emissions of ruminant animals, such as cattle, significantly contribute to GHGEs, exacerbating climate change.

Dietary patterns and their environmental impact are inconsistent across regions and sociodemographic groups.^9^ Cultural preferences, economic factors and social dynamics significantly shape dietary choices and consumption patterns.^10–12^ Previous research from country specific studies has suggested how variations in dietary patterns between European countries may result in different environmental footprints. For instance, a lower diet related GHGE has been found in Mediterranean countries like Spain^13^ and Italy^9^ when compared with central European countries such as France^14^ or The Netherlands.^15^

The European Union (EU) has set a target to reduce GHGE by at least 55% by 2030 comparing with 1990 levels, and agriculture is a leading contributor of GHGE in Europe.^16^ Understanding the variation in dietary patterns of the European population and associated environmental impacts is essential for the development of targeted interventions and policies that promote sustainable diets while considering the unique context of different populations. However, existing studies that examined individual food consumption are predominantly limited by analysis within few single European countries.^13–15^^,^^17–19^ Hence, based on the latest population-based and harmonised dietary survey data obtained from the European Food Safety Authority, this study aims to quantify the environmental impact of individual dietary patterns in 10 countries from all areas of Europe as well as to present a cross-country comparison of dietary patterns and their associated environmental impact stratified by socio-demographic factors.

## Methods

### Study population (EFSA)

We analyzed pooled cross-sectional national dietary records of adults in 10 European countries obtained from the European Food Safety Authority (EFSA) Comprehensive European Food Database. This dataset formed part of the EU Menu Project^20^ designed for the collection of harmonized dietary data across EU member states. We used the latest data available for 15 surveys from 10 EU Member States (16 894 subjects), collected through 24-, 48-, or 72-hour dietary records/recalls over the 2010–18 period (Supplementary Appendix SA1). Data from institutionalized participants and those aged under 18 or above 79 years were excluded from the analysis. Surveys were conducted year-round, encompassing all four seasons, and ensured a balanced proportional representation of data collection during weekdays and weekends.

All food and beverage items consumed by the survey participants were coded using the FoodEx2 classification system, created by the EFSA.^21^^,^^22^ It provides a standardized system to classify and categorize food items, allowing for consistent and harmonized reporting of dietary intake data from different surveys. The classification system includes various levels of detail, ranging from broad food groups (fruit and fruit products) to specific individual food items (apples).

### Environmental impact of individual diets

The environmental impact data associated with each food/beverage item was obtained from the publicly available SHARP Indicators Database (SHARP-ID).^23^ The SHARP-ID contains GHGE and LU data for 957 foods coded using FoodEx2. Environmental impact assessment relied on attributional life cycle analysis,^24^ which followed an internationally accepted standardized methodology in accordance with ISO14040 and 14044:2006.^2^ The GHGE and LU estimates of the 957 foods were obtained from life cycle inventory data of 182 primary products, which integrated information on food production, trade, and transport. The GHGE estimates incorporated carbon dioxide (CO_2_) emissions resulting from fossil fuel usage, methane (CH_4_) emissions generated during cattle rearing and certain crop cultivation, as well as nitrous oxide (N_2_O) emissions released from fertilizers, manure and ploughing of grasslands.^2^^,^^9^ The LU estimates were based on the surface area required for food production, accounting for conventional agricultural practices.^2^^,^^9^ The GHGE and LU data in the SHARP-ID had further accounted for the food quantity, edible portion, cooking losses and gains, food losses and waste. Due to data limitations, the contributions of industrial food processing (e.g. grinding, cutting, centrifuging and washing), storage, and transportation from retail to the home were excluded from the assessment.

The GHGE estimates in the SHARP-ID were provided in kilograms of food eaten of carbon dioxide equivalents (kgCO_2_-eq), and LU as square metres year (m^2^*year).

We linked each food item as recorded in the EU Menu dietary consumption data to its corresponding GHGE and LU values provided in the SHARP-ID.^9^^,^^22^ Assuming a homogeneous European market, a single value for GHGE and LU was assigned to each food item. This value was then applied to the food intake data of the ten countries included in the study. In cases where individual food items from the surveys were not listed in the SHARP-ID database, the GHGE and LU values were attributed based on the mean values of the corresponding food group (for approximately 5.1% of the total food items). However, no GHGE or LU data were available in the SHARP-ID database for four specific food groups: food additives, vitamin supplements, condiments and herbs and spices (accounting for roughly 4.0% of the total number food items). Hence, these four food groups were excluded from the analysis. Nevertheless, these food items are consumed in small quantities in National surveys, suggesting their impact on study findings are likely minimal.

Total diet-associated GHGE and LU were calculated for each individual by the sum of their values from all the grams of foods consumed, and averaged across multiple days to provide outcome measures relevant for daily food intake.

### Covariates

Covariates included in the analysis were gender, age (stratified into four categories: 18–35, 36–50, 51–65 and 66–79), educational level (classified as primary education: up to 9 years of education, secondary education: up to 12 years of education, and tertiary education: more than 12 years of education), and whether any special diet was consumed (classified as normal diet, vegetarian diet, and other diets, including slimming diets and diets related to health conditions).

Participants with missing education data were excluded from subsequent analyses. This applied to 8, 95 and 347 participants from Cyprus, Latvia and Austria, respectively. Dietary surveys from France only provided participants’ age in two broad groups (adults or elderly). Furthermore, education data were not made available by EFSA for dietary surveys from France, Belgium and Greece; and self-identified diet type were not made available for dietary surveys from Spain, Cyprus, Greece, Latvia and The Netherlands. Therefore, no analysis was conducted for these subcategories.

### Statistical analysis

Descriptive statistics were computed for the mean level of diet-related GHGE and LU overall and for each of the 10 EU countries. These were further divided into eight largest food groups where the mean dietary consumption (in grams/day and also as a proportion of total food intake) and the mean GHGE and LU (in kgCO_2_-eq or m^2^*year/day and also as a proportion of total GHGE or LU from diet) were calculated for each of the following: fish and seafood, fruit, grains, meat, dairy, beverages, vegetables, oils and fats and miscellaneous (e.g. confectionary, seasoning sauces). Furthermore, we computed the mean level of diet-related GHGE and LU for population subgroups of gender, age, education and diet type, standardized by participants’ total daily food intake and expressed as kgCO_2_-eq or m^2^*year/kg of food consumed. The subgroups analysis was performed per kilogram of food consumed per day to assess the relative quantities of consumption and the environmental impact of different foods and food groups.^3^ While the unstandardized measures provide the best approximation of total environmental impact associated with European diets, the standardized measures facilitate the comparison between population subgroups accounting for the quantities of food required/consumed. Multivariable linear regression models were used to compare participant’s diet-associated GHGE and LU (as standardized by participants’ total daily food intake) between countries and subgroups of gender, age, education and diet type. We also analyzed the contributions of major food groups to the mean daily total of GHGE and LU for each country.

## Results

A total of 15 498 participants from 10 European countries were included in this study. Number of participants ranged from 510 in Cyprus to 3073 in Estonia. Table 1 shows notable variations in the distributions of gender, age, and educational level among the analyzed countries. There were more female than male participants (59.5% vs. 40.5%) in all countries especially in Estonia. In most countries, around one third of participants fell within the 18–35 age range, while in Slovenia and Greece, this group constituted less than one fifth of the population. Across countries, most survey participants completed tertiary education, ranging from 50.3% to 89.5%. Furthermore, most people followed a normal diet without any restrictions (79.8% or above).

**Table 1:** Baseline characteristics of participants from EFSA food databases

Country	Total (%)	Estonia (%)	Latvia (%)	Austria (%)	Belgium (%)	France (%)	Netherlands (%)	Greece (%)	Cyprus (%)	Slovenia (%)	Spain (%)
*N*	15 498	3073	1505	2488	1269	2195	2147	519	510	841	951
Gender											
Male	6274 (40.5)	901 (29.3)	700 (46.5)	800 (32.1)	609 (47.9)	945 (43.1)	1071 (49.8)	258 (49.7)	183 (35.9)	401 (47.7)	408 (42.9)
Female	9220 (59.5)	2172 (70.7)	805 (53.5)	1688 (67.9)	660 (52.1)	1250 (56.9)	1076 (50.2)	261 (50.3)	327 (64.1)	440 (52.3)	543 (57.1)
Age groups											
18–35	4822 (36.3)	1099 (35.8)	476 (31.6)	1,291 (51.9)	527 (41.4)	^a^	693 (32.2)	98 (18.8)	222 (43.4)	124 (14.7)	292 (30.7)
36–50	3251 (24.1)	792 (25.8)	364 (24.1)	796 (31.9)	389 (30.6)		418 (19.4)	85 (16.3)	54 (10.5)	117 (13.9)	236 (24.8)
51–65	2797 (20.9)	713 (23.2)	356 (23.7)	401 (16.1)	353 (27.8)		397 (18.4)	98 (18.8)	77 (15.1)	207 (24.5)	195 (20.5)
66–79	2351 (18.7)	469 (15.2)	308 (20.5)	0 (0)	0 (0)		639 (29.7)	158 (45.9)	157 (30.9)	393 (46.7)	227 (23.9)
Education											
Primary	299 (2.9)	3 (0.1)	12 (0.7)	75 (3.0)	^b^	^b^	87 (4.0)	^b^	90 (17.5)	17 (2.0)	15 (1.5)
Secondary	1728 (15.3)	319 (10.3)	111 (7.3)	114 (4.5)			300 (13.9)		161 (31.5)	395 (47.0)	328 (34.5)
Tertiary	9264 (79.8)	2751 (89.5)	1334 (88.7)	2125 (85.4)			1760 (81.9)		257 (50.3)	429 (50.9)	608 (63.9)
Diet											
Normal diet	8333 (85.1)	2585 (84.1)	^b^	1986 (79.8)	1015 (79.9)	2009 (91.5)	^b^	^b^	^b^	738 (87.7)	^b^
Vegetarian diet	155 (1.5)	25 (0.8)		95 (3.8)	8 (2.5)	21 (0.9)				6 (0.7)	
Other diets^c^	1378 (13.3)	463 (15.8)		406 (16.3)	247 (19.4)	165 (7.5)				97 (11.5)	

a France only provides data regarding the two age groups (adults or elderly) and not in relation to the ages of each individual.

b No data available.

c Includes slimming diet and diets related to health conditions.

Consumption of beverages was the largest contributor to average daily food intake representing approximately half (40.6–54.1%) of total gram intake except for three countries (Supplementary Appendix SA3). Spain and Greece had a more balanced dietary pattern with most of their daily food intake sourced more evenly from beverages, dairy and vegetables. Slovenia was similar but their dietary patterns were largely based on beverages, grains and fruits. Moreover, Estonia, Spain, Greece and Slovenia had a larger proportion (>10%) of their diet sourced from fruits than other European countries. Vegetable consumption was highest in Greece, followed by Spain and Cyprus. Meat consumption was similar and ranged from 4.8% to 8.6% across countries except for Slovenia that showed the highest intake (10.7%). Fish and seafood consumption were highest in Spain (3.4%) and Greece (2.4%) but lower than 2% of total diet in other countries.

**Figure 1:**
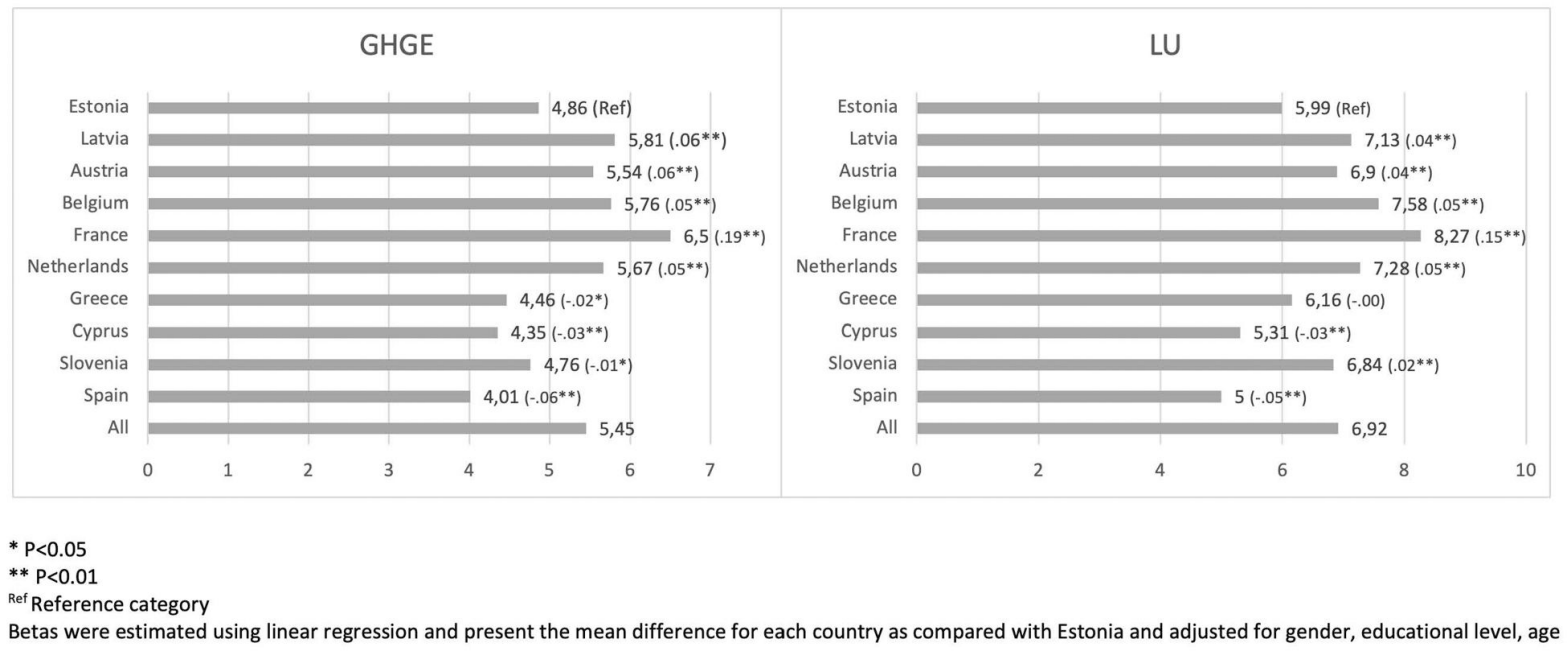
Comparison of mean Diet-related GHGE (kgCO2eq)/day and LU (m2*year)/day between 10 European countries (Beta). *P < 0.05; **P < 0.01; ^Ref^reference category. Betas were estimated using linear regression and present the mean difference for each country as compared with Estonia and adjusted for gender, educational level and age

Average dietary-associated GHGE and LU per capita varied significantly across countries (figure 1). Spain showed the lowest mean GHGE and LU per capita with 4.0 kgCO_2_eq/day and 5.0 m^2^year/day, respectively. In contrast, France (6.5 kgCO_2_eq/day; 8.2 m^2^year/day), Belgium (5.7 kgCO_2_eq/day; 7.5 m^2^year/day) and Latvia (5.8 kgCO_2_eq/day; 7.1 m^2^year/day) exhibited the highest values per capita. Supplementary Appendix SA2 shows the average of GHGE and LU of a daily diet by major food groups overall and in each country. Among the 10 European countries, dietary intake of meat and dairy products were on average the biggest contributor responsible for more than half of the mean daily GHGE and about two-thirds of LU. Meat and meat products accounted for 2.1 kgCO_2_eq or 40% of the total GHGE and dairy products contributed to 18.2% (0.9 kgCO_2_eq) of the total GHGE. Regarding LU, meat with and meat products was responsible for 53.1% (3.6 m^2^*year/day) and dairy products accounted for 13.7% (0.9 m^2^*year/day) of the total of LU. Yet, meat and meat products constituted for just an average of 6.3% of the total grams consumed (including beverages) by our sample (Supplementary Appendices SA3 and SA4).

In the analysis conducted within countries, meat consistently emerged as the primary environmental contributor across all countries. However, for GHGE and LU footprints, dairy products were not universally the second-highest contributors in all analyzed countries. Specifically, in Estonia (1.0 kgCO_2_eq/day; 0.9 m^2^*year/day) and Slovenia (0.4 kgCO_2_eq/day; 0.8 m^2^*year/day), beverages and grains/grain-based products, respectively, took the position of the second-highest contributors. Meanwhile, for Spain (0.7 m^2^*year/day), France (1.1 m^2^*year/day), and Belgium (0.9 m^2^*year/day), grains and grain-based products were the second highest contributors only for LU.

Among different demographic and dietary groups (tables 2 and 3), there were significant variations in diet-related GHGE and LU per kilogram of food consumed. This subgroup analysis was standardized per kilogram of food consumed per day to evaluate the relative quantities of consumption and the environmental impact of various foods and food groups. Females exhibited lower GHGE [2.6 kgCO_2_eq/day, *B* = −0.08 (SE = 0.01), *P* < 0.01] compared with males. This trend was consistent in almost all European countries except for Greece and Spain, where no significant differences were observed in diet-related GHGE. The older population aged 66–79 years showed lower mean GHGE [2.6 kgCO_2_eq/day, *B* = −0.03 (SE = 0.02), *P* < 0.01] compared with the youngest age group. These findings were consistently observed in Estonia, Spain, Cyprus and Greece. For Slovenia, no significant differences were found between age groups. In the pooled analysis, individuals with secondary education had a higher mean GHGE [2.7 kgCO_2_eq/day, *B* = 0.02 (SE = 0.02), *P* < 0.01] compared with those with higher or lower education levels. However, within country analysis showed no major differences between mean GHGE by education level, except for individuals with secondary education in Austria [3.1 kgCO_2_eq/day, *B* = 0.04 (SE = 0.10), *P* < 0.01] and Slovenia [3.6 kgCO_2_eq/day, *B* = 0.05 (SE = 0.10), *P* < 0.05], and individuals with primary education in Latvia [3.5 kgCO_2_eq/day, *B* = 0.04 (SE = 0.24), *P* < 0.01]. And finally, Individuals following vegetarian diets (as compared with normal diet) had a significantly lower GHGE [1.7 kgCO_2_eq/day, *B* = −0.07 (SE = 0.05), *P* < 0.01] overall and within each European countries where diet type data were available.

**Table 2:** Mean diet-related GHGE (kgCO2eq) per kg food per day (SD), and relationship between sociodemographic factors and GHG (Beta) (SE), by country

		Male (ref)	Female	18–35 (ref)	36–50	51–65	66–79	Primary	Secondary	Tertiary (ref)	Normal diet (ref)	Vegetarian diet	Other Diets	Total
**Estonia**	Mean (SD)	2.68 (1.14)	2.40 (1.08)	2.59 (1.19)	2.39 (1.00)	2.44 (1.10)	2.45 (1.05)	1.78 (0.82)	2.61 (1.61)	2.47 (1.47)	2.51 (1.09)	1.75 (1.14)	2.39 (1.17)	2.48 (1.10)
	Beta (SE)^c^		−0.112^b^(0.03)		−0.075^b^(0.03)	0.058^b^(0.03)	−0.046^b^(0.04)	−0.018(0.44)	0.020(0.04)			−0.062^b^(0.15)	−0.029^a^(0.03)	
**Latvia**		3.14 (1.20)	2.68 (1.13)	2.82 (1.20)	2.85 (1.18)	3.00 (1.16)	2.92 (1.18)	3.52 (1.46)	2.99 (1.10)	2.88 (1.19)				2.89 (1.18)
			−0.198^b^(0.04)		0.018(0.05)	0.074^b^(0.05)	0.030(0.06)	0.048^b^(0.24)	0.007(0.08)					
**Austria**		3.05 (1.69)	2.73 (1.48)	2.95 (1.71)	2.74 (1.38)	2.64 (1.30)		2.70 (1.73)	3.11 (1.61)	2.81 (1.53)	2.89 (1.53)	1.83 (0.93)	2.79 (1.69)	2.83 (1.56)
			−0.107^b^(0.04)		−0.066^b^(0.04)	−0.081^b^(0.06)		−0.002(0.12)	0.040^b^(0.10)			−0.134^b^(0.11)	−0.019(0.05)	
**Belgium**		2.72 (1.58)	2.49 (1.38)	2.67 (1.43)	2.53 (1.55)	2.58 (1.49)					2.66 (1.52)	0.92 (0.95)	2.39 (1.34)	2.60 (1.49)
			−0.078^b^(0.05)		−0.043^a^(0.07)	0.025(0.07)						−0.063^b^(0.37)	−0.062^b^(0.07)	
**France**		3.08 (1.78)	2.78 (1.58)								2.93 (1.65)	1.80 (0.87)	2.76 (1.65)	2.91 (1.67)
			−0.089^b^(0.04)									−0.062^b^(0.21)	−0.025^a^(0.07)	
**Netherlands**		2.33 (1.06)	2.18 (0.99)	2.29 (1.11)	2.24 (1.04)	2.17 (0.89)	2.29 (1.00)	2.36 (0.98)	2.28 (0.98)	2.25 (1.03)				2.26 (1.02)
			−0.074^b^(0.03)		−0.016(0.04)	0.043^b^(0.04)	−0.000(0.04)	0.019(0.08)	0.017(0.04)					
**Greece**		3.56 (2.04)	3.80 (2.43)	3.96 (2.24)	3.96 (2.27)	3.82 (2.67)	3.40 (2.02)							3.68 (2.25)
			0.051(0.13)		0.002(0.23)	−0.024(0.22)	−0.121^b^(0.19)							
**Cyprus**		2.44 (1.55)	2.29 (1.43)	2.51 (1.51)	2.36 (1.39)	2.25 (1.32)	2.14 (1.50)	2.41 (1.20)	2.13 (1.47)	2.46 (1.55)				2.34 (1.47)
			−0.080^b^(0.08)		−0.032(0.12)	0.066^a^(0.12)	−0.107^b^(0.12)	−0.007(0.10)	−0.047(0.11)					
**Slovenia**		3.63 (2.21)	2.92 (1.78)	3.31 (1.81)	3.41 (2.24)	3.31 (2.04)	3.17 (2.01)	3.39 (1.94)	3.36 (2.12)	3.16 (1.93)	3.28 (2.05)	1.68 (0.84)	3.18 (1.85)	3.26 (2.02)
			−0.175^b^(0.09)		0.009(0.18)	−0.024(0.16)	−0.063(0.14)	0.026(0.35)	0.056^a^(0.10)			−0.061^b^(0.58)	−0.011(0.15)	
**Spain**		2.96 (1.39)	2.92 (1.42)	3.24 (1.50)	2.91 (1.31)	2.81 (1.40)	2.69 (1.33)	2.32 (0.77)	2.86 (1.39)	3.00 (1.43)				2.94 (1.41)
			−0.025(0.06)		−0.098^b^(0.08)	0.120^b^(0.09)	−0.157^b^(0.09)	−0.036(0.26)	−0.009(0.07)					
**All** ^d^		2.90 (1.58)	2.61 (1.42)	2.81 (1.55)	2.64 (1.37)	2.65 (1.42)	2.69 (1.51)	2.54 (1.35)	2.75 (1.56)	2.63 (1.32)	2.85 (1.54)	1.78 (0.95)	2.72 (1.59)	2.73 (1.49)
			−0.087^b^(0.01)		−0.030^b^(0.02)	−0.031^b^(0.02)	−0.035^b^(0.02)	−0.010^a^(0.05)	0.027^b^(0.02)			−0.079^b^(0.05)	−0.022^b^(0.02)	

a *P* < 0.05.

b *P* < 0.01.

c Betas adjusted for gender, educational level, age.

d Betas adjusted for gender, educational level, age, country.

**Table 3:** Mean diet-related LU (m2^*^year) per Kg food per day (SD), and relationship between sociodemographic factors and LU (Beta) (SE), by country

		Male (ref)	Female	18–35 (ref)	36–50	51–65	66–79	Primary	Secondary	Tertiary (ref)	Normal diet (ref)	Vegetarian diet	Other Diets	Total
**Estonia**	Mean (SD)	3.49 (1.80)	2.93 (1.67)	3.27 (1.88)	2.96 (1.57)	3.01 (1.71)	3.02 (1.63)	2.40 (2.28)	3.47 (2.58)	3.05 (2.11)	3.14 (2.47)	1.66 (1.16)	2.93 (2.62)	3.09 (1.73)
	Beta (SE)^c^		−0.142^b^(.04)		0.073^b^(0.05)	0.067^b^(0.05)	−0.058^b^(0.06)	−0.010(0.69)	0.053^b^(0.07)			−0.076^b^(0.24)	−0.031^a^(0.06)	
**Latvia**		4.02 (1.89)	3.18 (1.68)	3.54 (1.86)	3.55 (1.86)	3.66 (1.77)	3.53 (1.80)	4.34 (2.58)	3.71 (1.58)	3.55 (1.84)				3.57 (1.83)
			−0.230^b^(0.06)		0.013(0.08)	0.040^a^(0.09)	−0.004(0.09)	0.043^b^(0.37)	0.011(0.12)					
**Austria**		4.07 (2.76)	3.40 (2.38)	3.83 (2.80)	3.44 (2.19)	3.29 (2.16)		3.52 (2.90)	4.04 (2.62)	3.57 (2.48)	3.71 (2.50)	2.14 (1.24)	3.52 (2.81)	3.62 (2.53)
			−0.136^b^(0.07)		−0.078^b^(0.08)	−0.089^b^(0.10)		0.005(0.20)	0.040^b^(0.17)			−0.123^b^(0.18)	−0.022(0.09)	
**Belgium**		3.74 (2.52)	3.25 (2.18)	3.67 (2.32)	3.35 (2.46)	3.37 (2.30)					3.58 (2.40)	1.80 (1.10)	3.14 (2.15)	3.49 (2.36)
			−0.103^b^(0.09)		0.061^b^(0.11)	0.054^b^(0.11)						−0.060^b^(0.58)	−0.061^b^(0.11)	
**France**		4.06 (2.85)	3.52 (2.44)								3.78 (2.64)	1.99 (1.01)	3.59 (2.69)	3.75 (2.64)
			−0.101^b^(0.06)									−0.063^b^(0.33)	−0.018(0.12)	
**Netherlands**		3.11 (1.72)	2.75 (1.63)	3.04 (1.85)	2.92 (1.74)	2.74 (1.41)	2.92(1.61)	3.23 (1.64)	3.02 (1.66)	2.90 (1.69)				2.93 (1.69)
			−0.110^b^(0.05)		−0.027(0.07)	0.069^b^(0.07)	−0.037^a^(0.06)	0.030^a^(0.13)	0.039^b^(0.07)					
**Greece**		4.98 (3.44)	5.29 (4.18)	5.57 (3.70)	5.61 (3.88)	5.43 (4.77)	4.66 (3.37)							5.14 (3.83)
			.038^b^(0.23)		0.004(0.40)	−0.014(0.38)	−0.117^b^(0.32)							
**Cyprus**		3.16 (2.43)	2.83 (2.16)	3.13 (2.50)	2.99 (2.20)	2.89 (2.30)	2.72 (2.41)	2.91 (1.77)	2.69 (2.39)	3.13 (2.36)				2.95 (2.27)
			−0.092^b^(0.12)		0.018(0.19)	−0.045(0.18)	−0.086^a^(0.18)	−0.026(0.16)	−0.043(0.17)					
**Slovenia**		5.38 (3.37)	4.10 (2.85)	4.69 (2.88)	4.96 (3.60)	4.81 (3.41)	4.59 (3.36)	5.30 (3.55)	4.93 (3.54)	4.48 (3.13)	4.73 (3.38)	2.29 (1.26)	4.67 (3.09)	4.71 (3.34)
			−0.190^b^(0.16)		0.018(0.29)	−0.013(0.26)	−0.051(0.24)	0.044(0.58)	0.073^b^(0.16)			−0.054^b^(0.95)	−0.004(0.25)	
**Spain**		3.88 (2.33)	3.66 (2.33)	4.30 (2.52)	3.75 (2.18)	3.45 (2.30)	3.32 (2.09)	2.79 (1.32)	3.65 (2.29)	3.83 (2.36)				3.75 (2.33)
			−0.061^b^(0.10)		−0.100^b^(0.14)	−0.154^b^(0.15)	−0.184^b^(0.15)	−0.024(0.43)	0.011(0.11)					
**All** ^d^		3.87 (2.55)	3.29 (2.25)	3.66 (2.50)	3.38 (2.20)	3.38 (2.30)	3.47 (2.41)	3.28 (2.28)	3.69 (2.58)	3.33 (2.11)	3.69 (2.47)	2.02 (1.16)	3.51 (2.62)	3.52 (2.40)
			−0.110^b^(0.02)		−0.030^b^(0.03)	−0.036^b^(0.03)	−0.043^b^(0.03)	0.005(0.09)	−0.048^b^(0.04)			−0.076^b^(0.09)	−0.019^b^(0.04)	

a *P* < 0.05.

b *P* < 0.01.

c Betas adjusted for gender, educational level, age.

d Betas adjusted for gender, educational level, age, country.

LU patterns mirrored GHGE findings, with notable gender differences in Greece. Specifically, females showed significantly higher LU [5.2 m^2^*year/day, *B* = −0.03 (SE = 0.23), *P* < 0.01] compared with males.

## Discussion

In this large, population-based cross-sectional study of environmental impacts associated with dietary patterns of adults from 10 European countries, the mean dietary-associated impact of GHGE and LU per capita were found highest in France, Belgium and Latvia. Conversely, it was the lowest in Spain, Cyprus and Greece. While meat and meat product consumption were understandably the largest contributor of GHGE and LU impact for all European populations, the comparatively lower overall GHGE and LU impact observed in Spain and Greece may be explained by a more diverse dietary pattern with contribution of major food groups spread more evenly between fruits, vegetables, grains, dairy and beverages. France, Belgium and Cyprus had similar dietary patterns predominantly sourced from beverages, followed by vegetables and dairy. However, the mean total food intake per capita were much higher in France and Belgium than Cyprus (2351, 2335 and 2058 g/day, respectively). Furthermore, our findings suggest a commonly higher dietary-associated GHGE and LU as characterized by male, younger persons and non-vegetarians among the European populations. However, we did not find major differences in dietary-associated environmental impact between education level subgroups.

These results are consistent with recent findings from a comparable study of four European countries which used EFSA and SHARP-ID databases.^9^ In that study, France emerged as the nation with the highest levels of GHGE and LU footprints. Moreover, it showed that a Mediterranean country, namely Italy, had a generally lesser environmental impact. The type of foods chosen within a food group and relative consumption quantities may also play a part in these results, particularly if the Mediterranean countries consume a lower proportion of ruminant meat that are associated with the highest environmental impact.^25^^,^^26^ This aligns with the evidence that links Mediterranean-style diets to lower GHGE and LU footprints.^18^^,^^27^ It illustrates how transitioning to a plant-based diet, which is also associated with improved health outcomes,^28^ can diminish our daily environmental impact.

Our calculated average of diet-related GHGE spanned from 4.0–6.5 kgCO_2_eq/day within the ten European countries studied. These figures match those recorded in France from a similar study on four European countries (6.0 kgCO_2_eq/day).^9^ However, they are 58% higher than the result (4.1 kgCO_2_eq/day) from an earlier investigation.^14^ Additionally, our estimations were comparable to those previously reported in the Netherlands (5.3 kgCO_2_eq/day),^15^ and they were 29% higher than the values for Spain (3.1 kgCO_2_eq/day).^13^

Making a direct comparison of daily footprints with other studies is hindered by variations in the foundational Life Cycle Assessment (LCA) methodology and survey samples. First, these studies use different dietary surveys and different energy density outcome measures. While our analysis evaluated the relative consumption quantities and their environmental impact per kilogram of daily food intake, other studies standardized GHGE and LU based on calories intake (e.g. 2000 kcal).^9^^,^^19^ It is worth noting that energy measurements can be skewed by the presence of low-calorie foods and drinks, whereas gram-based metrics are weighted towards beverage intake. Second, while the SHARP-ID database uses a standardized method to derive GHGE and LU values across all countries, discrepancies may exist between countries due to factors such as intensive vs. extensive animal production systems, and greenhouse vs. open-field methods for animal feed and crop growth. Supply chain considerations, including domestic vs. foreign production, transportation methods and distances, packaging and preparation methods, and food losses and waste, can also contribute to these variations.^29^ Additionally, for most food items, the main source of environmental impact lies within the agricultural phase, which includes crop production and animal breeding that are greatly influenced by different management practices between countries.^30^ Although the SHARP-ID database used in this study assumes a homogeneous European market and does not consider country-specific dietary footprints from different agricultural systems, its standardized coding of food items facilitated by the use of FoodEx2 allows for the direct comparison of dietary patterns among harmonized and representative dietary survey data obtained from EFSA. Moreover, since all included countries belong to the European Union, where they share a common agricultural policy,^31^ and European Common Market and Trade policy (free movement of goods, services, capital and persons in a single EU internal market),^32^ we do not expect substantial discrepancies between the analyzed countries. Further research includes examination of other environmental indicators such as freshwater use, eutrophication or biodiversity loss to present a more complete picture of diet-related environmental footprints between populations.

Significant variations in diet-related GHGE and LU per kilogram of food consumed were observed among different demographic and dietary groups. Specifically, women exhibited lower GHGE and LU compared with men after adjusting for grams consumed, age, education and country, indicating potential gender-related differences in environmental impact. These findings are in line with evidence that points to a higher footprint among men,^9^^,^^15^ even after adjusting for calories or grams consumed by men. It may be partially explained by differences in diet choices among women found in previous studies,^9^ such as higher consumption of fruits and vegetables, and lower intake of red and processed meat. There were some noteworthy exceptions to highlight. Specifically, Spain and Greece, both of which had some of the lowest GHGE levels among the countries, showed no apparent differences in GHGE between genders.

Lower GHGE and LU observed among the older population compared with other age groups may be attributed to similar factors.^33^ Still, it is unclear if these discrepancies are linked to generational shifts in dietary patterns or if they are due to changes in eating behaviours as people age. Furthermore, people with secondary education showed a slightly higher GHGE and LU footprints than both highest educated and lowest educated individuals. This can also be partly explained by higher consumption of fruits and vegetables, and lower intake of meat by the higher educated people.^9^^,^^33^ The findings concerning individuals with primary education should be interpreted cautiously, given that this group comprises only approximately 3% of our sample and is largely inconsistent across the countries under analysis. Furthermore, the variations in diet-related GHGE and LU among different education subgroups were generally small and inconsistent across the countries analyzed.

Dietary consumption data were sourced from the EU Menu project, ensuring high-quality, representative and harmonized individual dietary records across EU member states. The mean portion sizes (grams) may differ across countries due to population characteristics, adjusted for in our main findings. For instance, Spain and Greece indeed had comparatively lower mean (unadjusted) portion sizes for beverages. However, Spain ranked second in milk/dairy portions and third in fruit portions, while Greece had the largest portions for fats/oils and substantial portions for fish, seafood and vegetables compared with other countries.

Finally, when considering the combined data from the 10 European countries, it is noteworthy that meat and dairy intake contributed to over half of the total GHGE and approximately two-thirds of the overall LU. Yet, this impact was derived from an average consumption of just 6.3% of the total grams consumed (including beverages) within our sample. Not surprisingly, individuals following vegetarian diets demonstrated lower GHGE and LU compared with those following non-vegetarian diets, highlighting once again the potential environmental benefits of adopting a mostly plant-based dietary patterns.^3^^,^^34^^,^^35^ These findings not only reinforce the well-established notion in the literature regarding the imperative for bold action to reduce red meat consumption in Europe but also highlight the significance of considering demographic and dietary factors in assessing the environmental impact of food consumption. It is worth point out that beyond meat intake, diet diversity and portion size also play an essential role in shaping environmental footprints. As seen in countries such as Spain and Greece, greater dietary diversity with a balanced contribution from major food groups like fruits, vegetables, grains and dairy can result in a reduced GHGE and LU impact. The contrast between countries with high environmental footprints such as France and Belgium and those with lower footprints like Cyprus show that reducing portion sizes can also lead to a lower environmental impact. These insights can be invaluable in formulating targeted interventions and policies aimed at promoting more sustainable food consumption patterns.

## Supplementary Material

ckae088_Supplementary_Data

## Data Availability

The environmental impact data underlying this article are available in SHARP Indicators Database, at https://lifesciences.datastations.nl/dataset.xhtml?persistentId=doi:10.17026/dans-xvh-x9wz. The EFSA Comprehensive European Food Consumption Databases were sourced from the EU Menu project and can be requested at https://www.efsa.europa.eu/en/data-report/food-consumption-data. Key points The environmental impact of food consumption varies across countries, socioeconomic groups and dietary patterns. Highest dietary-associated GHGE and LU impacts per capita were in France, Belgium, and Latvia; lowest in Spain, Cyprus, and Greece. Greater environmental impacts were found associated with dietary patterns of men, younger individuals, and those with secondary education. Despite representing a small portion of total food consumption, meat consumption had the largest environmental impact in all European populations, emphasizing the environmental benefits of plant-based eating. Diet diversity and portion size significantly influenced diet-related environmental footprints, suggesting the importance of balanced dietary choices for sustainability.
