# Supplementary material for: Environmental impact of dietary patterns in 10 European countries; a cross-sectional analysis of nationally representative dietary surveys
Source: Eur J Public Health. 2024 May 22;34(5):992–1000. doi: 10.1093/eurpub/ckae088 (PMC11430961; doi:10.1093/eurpub/ckae088)
Supplement: ckae088_Supplementary_Data [file ckae088_supplementary_data.zip › ckae088_Supplementary_Data/ejph-2023-10-om-0569-File003.docx]

Appendix 1 – Summary of included EU Menu surveys from 10 European countries obtained from the EFSA Food Consumption Database

| **Countries** | **Survey** | **Year** | **Dietary methods** | **Subjects** |
| --- | --- | --- | --- | --- |
| Estonia | National Dietary Survey Estonia | 2013-2015 | (2 days) Food records/24-hour dietary recall | 3,073 |
| Latvia | Latvian National Dietary Survey | 2012-2015 | (2 days) Food records/24-hour dietary recall | 1,611 |
| Austria | Austrian Study on Nutritional Status 2016 | 2016 | (2 days) Food record | 2,250 |
| Belgium | Belgium, national food consumption survey adults | 2014-2015 | (2 days) 24-hour dietary recall | 1,304 |
| France | The French national dietary survey (INCA3, 2014-2015) | 2014-2015 | (3 days) 24-hour dietary recall | 2,387 |
| Netherlands | Dutch National Food Consumption Survey 2012-2016 (DNFCS) | 2012-2017 | (2 days) Food records/24-hour dietary recall | 1,926 |
| Greece | Diet and Nutrition Survey 2014 - 2015 | 2014 - 2015 | (2 days) Food record | 513 |
| Cyprus | National dietary survey of CYPRUS 2014 - 2017 | 2014-2017 | (3 days) Food record | 736 |
| Slovenia | Slovenian national food consumption survey | 2017-2018 | (2 days) Food records/24-hour dietary recall | 846 |
| Spain | Spanish National dietary survey adults | 2012-2014 | (2 days) Food records/24-hour dietary recall | 1,862 |
