# Supplementary material for: Environmental impact of dietary patterns in 10 European countries; a cross-sectional analysis of nationally representative dietary surveys
Source: Eur J Public Health. 2024 May 22;34(5):992–1000. doi: 10.1093/eurpub/ckae088 (PMC11430961; doi:10.1093/eurpub/ckae088)
Supplement: ckae088_Supplementary_Data [file ckae088_supplementary_data.zip › ckae088_Supplementary_Data/ejph-2023-10-om-0569-File004.docx]

Appendix 2 – Contributions of major food groups to mean daily total (%) of GHGE (kgCO2eq) and LU (m2*year), by country

|  | **Vegetables and vegetable products** | **Fruit and fruit products** | **Grains and grain-based products** | **Fish, seafood, amphibians, reptiles** | **Meat and meat products** | **Milk and dairy products** | **Animal and vegetable fats and oils** | **Beverages** | **Miscellaneous*** |
| --- | --- | --- | --- | --- | --- | --- | --- | --- | --- |
|  | GHGE / % | GHGE / % | GHGE / % | GHGE / % | GHGE / % | GHGE / % | GHGE / % | GHGE / % | GHGE / % |
|  | LU / % | LU / % | LU / % | LU / % | LU / % | LU / % | LU / % | LU / % | LU / % |
| **Estonia** | 0,23 / 4,7% | 0,16 / 3,2% | 0,22 / 4,5% | 0,30 / 6,1% | 1,93 / 39,7% | 0,48 / 9,8% | 0,26 / 5,3% | 1,05 / 21,6% | 0.63 / 12.9% |
|  | 0,05 / 0,8% | 0,11 / 1,8% | 0,60 / 10,0% | 0,05 / 0,8% | 3,18 / 53,0% | 0,23 / 3,8% | 0,37 / 6,1% | 0,99 / 16;5% | 0.59 / 9.8% |
| **Latvia** | 0,25 / 4,3% | 0,12 / 2,1% | 0,39 / 6,7% | 0,35 / 6,3% | 2,35 / 40,5% | 1,10 / 19,0% | 0,28 / 4,8% | 0,51 / 8,8% | 0,87 / 15.0% |
|  | 0,06 / 0,8% | 0,07 / 1,0% | 0,85 / 11,9% | 0,05 / 0,7% | 3,82 / 53,6% | 1,06 / 14,9% | 0,34 / 4,8% | 0,20 / 2,8% | 0,83 / 11,6% |
| **Austria** | 0,32 / 5,8% | 0,14 / 2,6% | 0,61 /11,1% | 0,17 / 3,1% | 1,97 / 35,9% | 1,08 / 19,7% | 0,29 / 5,3% | 0,61 / 11,1% | 0,77 / 14,0% |
|  | 0,14 / 2,0% | 0,12 / 1,7% | 1,10 / 15,9% | 0,03 / 0,4% | 3,29 / 47,7% | 1,04 / 15,1% | 0,38 / 5,5% | 0,30 / 4,3% | 0,69 / 10.0% |
| **Belgium** | 0,22 / 3,9% | 0,09 / 1,5% | 0,48 / 8,4% | 0,32 / 5,6% | 2,65 / 46,6% | 0,78 / 13,7% | 0,19 / 3,3% | 0,65 / 11,4% | 0.71 / 12.4% |
|  | 0,08 / 1,1% | 0,06 / 0,7% | 0,91 / 13,2% | 0,06 / 0,7% | 4,44 / 58,6% | 0,75 / 9,9% | 0,36 / 4,7% | 0,39 / 5,1% | 0.76 / 10.2% |
| **France** | 0,23 / 4,2% | 0,12 / 2,2% | 0,65 / 11,9% | 0,45 / 8,2% | 2,70 / 49,5% | 1,09 / 20,0% | 0,33 / 6,0% | 0,64 / 11,7% | 0.73 / 11.3% |
|  | 0,13 / 1,6% | 0,09 / 1,9% | 1,12 / 13,5% | 0,08 / 0,9% | 4,55 / 55,0% | 1,05 / 12,7% | 0,50 / 6,0% | 0,31 / 3,7% | 0.67 / 8,1% |
| **Netherlands** | 0,22 / 3,9% | 0,09 / 1,6% | 0,50 / 8,8% | 0,21 / 3,7% | 2,15 / 38,0% | 1,07 / 18,9% | 0,16 / 2,8% | 0,91 / 16,1% | 1,11 / 19,6% |
|  | 0,10 / 1,4% | 0,07 / 1,0% | 1,00 / 13,7% | 0,04 / 0,5% | 3,58 / 49,2% | 1,00 / 13,7% | 0,38 / 5,2% | 0,50 / 6,9% | 0,97 / 13,4% |
| **Greece** | 0,24 / 5,4% | 0,10 / 2,2% | 0,50 / 6,7% | 0,29 / 6,5% | 1,93 / 43,3% | 0,97 / 21,7% | 0,14 / 3,1% | 0,16 / 3,6% | 0,24 / 5.3% |
|  | 0,06 / 1,0% | 0,18 / 2,9% | 0,88 / 11,9% | 0,02 / 0,3% | 3,29 / 53,4% | 0,97 / 15,7% | 0,52 / 8,4% | 0,10 / 1,6% | 0,28 / 4,5% |
| **Cyprus** | 0,29 / 6,7% | 0,11 / 2,5% | 0,36 / 11,2% | 0,29 / 6,7% | 1,58 / 36,7% | 0,88 / 20,5% | 0,13 / 3,0% | 0,41 / 9,5% | 0,44 / 10.2% |
|  | 0,07 / 1,3% | 0,10 / 1,8% | 0,70 / 14,3% | 0,02 / 0,3% | 2,58 / 48,6% | 0,85 / 16,0% | 0,42 / 7,9% | 0,16 / 3,0% | 0,50 / 9,4% |
| **Slovenia** | 0,27 / 5,7% | 0,13 / 2,7% | 0,43 / 9,1% | 0,17 / 3,5% | 2,73 / 57,5% | 0,42 / 8,8% | 0,19 / 4,0% | 0,32 / 6,7% | 0,44 / 9,2% |
|  | 0,05 / 0,7% | 0,09 / 1,3% | 0,87 / 12,7% | 0,03 / 0,4% | 4,56 / 66,7% | 0,40 / 5,8% | 0,34 / 5,0% | 0,15 / 2,2% | 0,45 / 6,5% |
| **Spain** | 0,15 / 3,8% | 0,31 / 7,8% | 0,38 / 9,7% | 0,47 / 11,9% | 1,60 / 40,7% | 0,75 / 19,1% | 0,10 / 2,5% | 0,21 / 5,3% | 0.29 / 7,3% |
|  | 0,06 / 1,2% | 0,11 / 2,2% | 0,70 / 14,0% | 0,04 / 0,8% | 2,67 / 53,4% | 0,69 / 13,8% | 0,30 / 6,0% | 0,16 / 3,2% | 0.37 / 7,4% |
| **All** | 0,24 / 4,4% | 0,12 / 2,2% | 0,47 / 8,6% | 0,30 / 5,5% | 2,21 / 40,7% | 0,99 / 18,2% | 0,24 / 4,4% | 0,57 / 10,5% | 0,71 / 13,0% |
|  | 0,09 / 1,3% | 0,09 / 1,3% | 0,90 / 13,0% | 0,05 / 0,7% | 3,68 / 53,1% | 0,95 / 13,7% | 0,39 / 5,6% | 0,29 / 4,1% | 0,67 / 9,6% |

* Includes sugar confectionery, starchy roots tubers, seasoning sauces, non-standard diets foods, legumes, nuts and seeds, kids food, eggs and composite dishes
