# Supplementary material for: Environmental impact of dietary patterns in 10 European countries; a cross-sectional analysis of nationally representative dietary surveys
Source: Eur J Public Health. 2024 May 22;34(5):992–1000. doi: 10.1093/eurpub/ckae088 (PMC11430961; doi:10.1093/eurpub/ckae088)
Supplement: ckae088_Supplementary_Data [file ckae088_supplementary_data.zip › ckae088_Supplementary_Data/ejph-2023-10-om-0569-File005.docx]

Appendix 3 – Daily mean (%) grams consumption for each food group, by country

|  | **Vegetables and vegetable products** | **Fruit and fruit products** | **Grains and grain-based products** | **Fish, seafood, amphibians, reptiles** | **Meat and meat products** | **Milk and dairy products** | **Animal and vegetable fats and oils** | **Beverages** | **Miscellaneous*** | **Total** |
| --- | --- | --- | --- | --- | --- | --- | --- | --- | --- | --- |
| **Estonia** | 165.6 / 8.0% | 228.6 / 11.1% | 157.2 / 7.7% | 26.9 / 1.2% | 130.9 / 6.3% | 302.5 / 14.8% | 18.9 / 0.8% | 831.8 / 40.7% | 177.4 / 8.6% | 2040.1 |
| **Latvia** | 197.7 / 9.6% | 146.6 / 7.1% | 173.8 / 8.4% | 30.9 / 1,4% | 177.4 / 8.6% | 202.1 / 9.8% | 13.5 / 0.6% | 833.9 / 40.6% | 275.4 / 13.4% | 2051.5 |
| **Austria** | 210.6 / 10.2% | 141.4 / 6.8% | 232.6 / 11.2% | 17.1 / 0,8% | 116.6 / 5.6% | 236.8 / 11.4% | 16.7 / 0.7% | 911.3 / 44.3% | 171.1 / 8.3% | 2054.5 |
| **Belgium** | 179.3 / 7.6% | 123.2 / 5.2% | 200.6 / 8.5% | 28.1 / 1,2% | 153.6 / 6.5% | 177.2 / 7.5% | 20.7 / 0.8% | 1264.2 / 54.1% | 187.9 / 8.0% | 2335.3 |
| **France** | 241.7 / 10.2% | 157.2 / 6.6% | 231.3 / 9.0% | 38.1 / 1.6% | 148.0 / 6.2% | 251.8 / 10.6% | 24.2 / 1.0% | 1072.8 / 45.6% | 186.4 / 7.9% | 2351.8 |
| **Netherlands** | 171.8 / 6.6% | 129.8 / 4.9% | 215.6 / 8.3% | 20.9 / 0,5% | 126.6/ 4.8% | 328.7 / 12.6% | 23.3 / 0.8% | 1354.2 / 52.2% | 218.8 / 8.4% | 2590.1 |
| **Greece** | 206.1 / 16.3% | 155.8 / 12.2% | 187.2 / 14.8% | 31.8 / 2,4% | 100.3 / 7.9% | 199.5 / 15.7% | 36.5 / 2.8% | 244.4 / 19.3% | 100.7 / 7.9% | 1262.6 |
| **Cyprus** | 221.7 / 10.7% | 132.0 / 6.4% | 164.7 / 7.9% | 30.6 / 1,4% | 131.6 / 6.3% | 216.3 / 10.5% | 27.3 / 1.3% | 981.0 / 47.6% | 152.8 / 7.3% | 2058.4 |
| **Slovenia** | 152.3 / 10.3% | 181.1 / 12.0% | 232.6 / 15.4% | 14.9 / 0,9% | 162.2 / 10.7% | 128.5 / 8.5% | 17.3 / 1.1% | 482.2 / 32.0% | 135.5 / 8.9% | 1506.1 |
| **Spain** | 146.3 / 10.7% | 177.2 / 12.9% | 149.3 / 10.9% | 47.2 / 3.4% | 104.6 / 7.6% | 317.7 / 23.2% | 19.7 / 1.4% | 285.4 / 20.9% | 115.8 / 8.4% | 1363.5 |
| **All** | 193.9 / 9.1% | 162.0 / 7.6% | 199.6 / 9.4% | 28.2 / 1.3% | 133.6 / 6.3% | 252.9 / 11.9% | 20.7 / 0.9% | 930.3 / 44.1% | 184.0 / 8.7% | 2084.2 |

* Includes sugar confectionery, starchy roots tubers, seasoning sauces, non-standard diets foods, legumes, nuts and seeds, kids food, eggs and composite dishes
