# Supplementary material for: Environmental impact of dietary patterns in 10 European countries; a cross-sectional analysis of nationally representative dietary surveys
Source: Eur J Public Health. 2024 May 22;34(5):992–1000. doi: 10.1093/eurpub/ckae088 (PMC11430961; doi:10.1093/eurpub/ckae088)
Supplement: ckae088_Supplementary_Data [file ckae088_supplementary_data.zip › ckae088_Supplementary_Data/ejph-2023-10-om-0569-File006.docx]

Appendix 4 – Food group contributions to total diet quantity (%) and to total diet-associated GHGE (%) and LU (%).
